# The Chimney Technique to Encounter Challenging Necks in Endovascular Aneurysm Repair: Past, Present...But is There Any Future Left?

**DOI:** 10.31083/j.rcm2306187

**Published:** 2022-05-25

**Authors:** Marco V. Usai, Konstantinos P. Donas

**Affiliations:** ^1^Department of Vascular Surgery, St. Franziskus Hospital, 48145 Münster, Germany; ^2^Department of Vascular and Endovascular Surgery, Research Vascular Center, Asklepios Clinic Langen, 63225 Langen, Germany

The chimney technique was introduced as a bail-out option to save inadvertently covered visceral arteries during EVAR [1]. As endovascular aortic surgery expanded its boundaries, the chimney technique was adopted in the treatment of complex aortic pathologies, mainly due to severe anatomical obstacles outside of instruction of use for fenestrated endografting.

After the publication of the PERICLES multicenter registry focusing on the use of the chimney technique in the treatment of juxta and pararenal aortic aneurysms, a clearer message was achieved bringing the chimney technique as a therapeutic option in the armamentarium of the vascular surgeon. Through this registry more points of view of this technique were put under the magnifying glass showing promising early and long-term results [2]. At mid-term overall ch-EVAR-related mortality was 2.2%. Freedom from primary and secondary type Ia endoleak/endotension were at 3 years was 93.0% and 98.0%, respectively. Primary and secondary chimney graft patency was 87.0% and 89.0%. Primary and secondary endovascular freedom from any endpoint at 3 years was 81.0% and 94.0% respectively [3].

Later on, after the publication of the PROTAGORAS 2.0 study a standard combination of the Endurant II endograft coupled with the Advanta V12/iCast with an aortic endograft oversizing of 30% demonstrated satisfactory mid-term results with low risk of persistent gutter-related endoleaks [4, 5].

Nevertheless, skepticism arouse among experts as the technique spread. In particular gutter related endoleaks are seen as an Achilles heel of this procedure, leading to difficult to treat type Ia endoleaks. However, the rate of such endoleaks remains low, as the majority of intraoperative eobserved gutters disappear with time.

Fenestrated aortic repair is a valid alternative. Nonetheless, reinterventions within 2 years after f-EVAR are common and in almost 40% of the treated patients, as observed in a large series from Sveinsson *et al*. [6]. Furthermore, fenestrated devices are not available in urgent situations, and this time to manufacturing and delivery of the device is sometimes too long and for some patients late due to rupture of their aneurysm in the meanwhile. Additionally, f-EVAR is not available in many countries in the world due to the very high costs and complexity in preoperative planning and deploying. Moreover, a certain number of anatomical factors such as stenosis, calcification and tortuosity of the iliac vessels, suprarenal angulation plays a remarkable role to search for alternatives than f-EVAR.

**Fig. 1.**
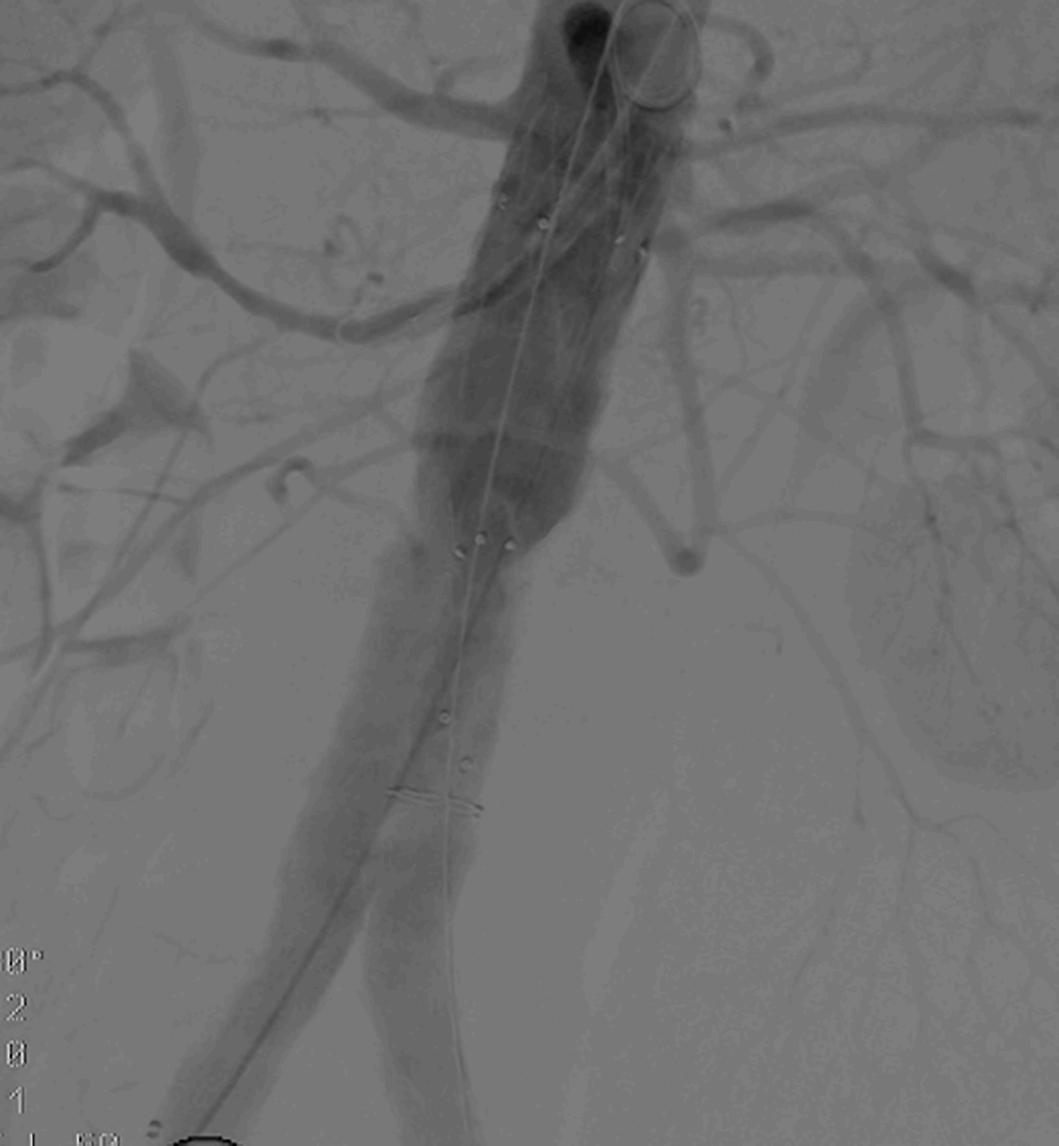
**Single ch-EVAR to preserve a low accessory right renal artery**.

Ch-EVAR is recognized as primary option in urgent conditions and as an alternative option in cases where f-EVAR is not indicated due to anatomical obstacles or not available as in many countries in the world, based on the European Guidelines of Vascular Surgery [7]. But also in a more specific analysis [8, 9], we see significant benefits and priority in the treatment by Ch-EVAR in also following indications:

- Octogenarians unfit for open repair with per se limited life expectancy, without the need to treat this group of patients with highly costs devices as in case of fenestrated endografting with 3 bridging devices for the visceral vessels [10].

- Accessory renal artery with perfusion of more than 40% of the kidney can be preserved with the ch-EVAR technique avoiding unnecessary loss of renal function [11] (Fig. 1).

- Younger patients with good life expectancy and without the necessity to deploy and involve bridging devices in the superior mesenteric artery having unknown patency over time and having also the risk of in-stent stenosis or occlusion in the most valuable target vessel of the aorta [12, 13].

- The technique is more comfortable for physicians with experience with EVAR compared to other options.

To our opinion Ch-EVAR has its stable place in the armamentarium of the vascular surgeon, however, very important is to highlight the standardization of this approach, the used devices and indications in order to have durable results.
